# Changes in beverage purchases following the announcement and implementation of South Africa’s Health Promotion Levy: an observational study

**DOI:** 10.1016/S2542-5196(20)30304-1

**Published:** 2021-04

**Authors:** Nicholas Stacey, Ijeoma Edoka, Karen Hofman, Elizabeth C Swart, Barry Popkin, Shu Wen Ng

**Affiliations:** SAMRC/Wits Centre for Health Economics and Decision Science – PRICELESS SA, Wits School of Public Health, Faculty of Health Sciences, University of the Witwatersrand, Johannesburg, South Africa; Department of Health Policy, London School of Economics and Political Science, London, UK; SAMRC/Wits Centre for Health Economics and Decision Science – PRICELESS SA, Wits School of Public Health, Faculty of Health Sciences, University of the Witwatersrand, Johannesburg, South Africa; SAMRC/Wits Centre for Health Economics and Decision Science – PRICELESS SA, Wits School of Public Health, Faculty of Health Sciences, University of the Witwatersrand, Johannesburg, South Africa; Department of Dietetics and Nutrition, University of the Western Cape, Cape Town, South Africa; DST/NRF Center of Excellence in Food Security, University of the Western Cape, Cape Town, South Africa; Department of Nutrition, Gillings School of Global Public Health, University of North Carolina, Chapel Hill, NC, USA; Carolina Population Center, University of North Carolina, Chapel Hill, NC, USA; Department of Nutrition, Gillings School of Global Public Health, University of North Carolina, Chapel Hill, NC, USA; Carolina Population Center, University of North Carolina, Chapel Hill, NC, USA

## Abstract

**Background:**

In 2016, South Africa announced an intention to levy a tax on sugar-sweetened beverages (SSBs). In 2018, the country implemented an SSB tax of approximately 10%, known as the Health Promotion Levy (HPL). We aimed to assess changes in the purchases of beverages before and after the HPL announcement and implementation.

**Methods:**

We used Kantar Europanel data on monthly household purchases between January, 2014, and March, 2019, among a sample of South African households (n=113 653 household-month observations) from all nine provinces to obtain per-capita sugar, calories, and volume from taxable and non-taxable beverages purchased before and after the HPL announcement and implementation. We describe survey-weighted means for each period, and regression-controlled predictions of outcomes and counterfactuals based on pre-HPL announcement trends, with bootstrapped 95% CIs, and stratify results by socioeconomic status.

**Findings:**

Mean sugar from taxable beverage purchases fell from 16·25 g/capita per day (95% CI 15·80–16·70) to 14·26 (13·85–14·67) from the pre-HPL announcement to post-announcement period, and then to 10·63 g/capita per day (10·22–11·04) in the year after implementation. Mean volumes of taxable beverage purchases fell from 518·99 mL/capita per day (506·90–531·08) to 492·16 (481·28–503·04) from pre-announcement to post announcement, and then to 443·39 mL/capita per day (430·10–456·56) after implementation. Across these time periods, there was a small increase in the purchases of non-taxable beverages, from 283·45 mL/capita per day (273·34–293·56) pre-announcement to 312·94 (296·29–329·29) post implementation. When compared with pre-announcement counterfactual trends, reductions in taxable beverage purchase outcomes were significantly larger than the unadjusted survey-weighted observed reductions. Households with lower socioeconomic status purchased larger amounts of taxable beverages in the pre-announcement period than did households with higher socioeconomic status, but demonstrated bigger reductions after the tax was implemented.

**Interpretation:**

The announcement and introduction of South Africa’s HPL were followed by reductions in the sugar, calories, and volume of beverage purchases.

**Funding:**

Bloomberg Philanthropies, International Development Research Centre, South African Medical Research Council, and the US National Institutes of Health.

## Introduction

Sub-Saharan Africa faces increasing issues of diet-related non-communicable diseases with rapidly rising intakes of sugar-sweetened beverages (SSBs) and other ultra-processed foods.^1-4^ South Africa, in particular, has high levels of burden related to these non-communicable diseases.^5,6^ Sugar, particularly from beverages, has been viewed as an important cause of increased risk of diabetes, hypertension, cardiovascular disease, and many common cancers.^7-10^

Many jurisdictions across the globe have used fiscal policy, namely taxation, as a major approach to curbing consumption of SSBs. Such taxes have been shown to be impactful when well designed and implemented.^11,12^ For example, in 2014, Mexico introduced a 1-peso-per-litre tax on beverages containing added sugar, resulting in a 6% reduction in purchased volume relative to pre-tax trends over the first year of the tax, and 7·6% reduction over the first 2 years of the tax.^13-15^ SSB tax policies implemented in other countries such as the UK and several subnational jurisdictions in the USA have also resulted in statistically significant reductions in SSB purchases.^16-23^

Given that SSBs are a heterogeneous set of products containing variations in sugar levels, taxing SSBs according to their sugar content more precisely targets the source of these products’ harms and implicitly incentivises beverage manufacturers to reduce the sugar content of their products.^24,25^ This strategy formed the basis of South Africa’s 2018 tax policy on SSBs—the Health Promotion Levy (HPL).^26^ A process to explore adopting the HPL began following its announcement in the late-February, 2016, budget speech. This was followed by a white paper published in June, 2016, reviewing evidence and making recommendations for a sugar-based tax to be levied at 0·028 South African rand (ZAR) per gram of sugar, a tax burden of approximately 20% of the per-litre price of the most popular soft drink.^24^

Proposed legislation containing the HPL was introduced to the South Africa National Assembly in April, 2017, with an extensive consultative period involving the sugar industry, beverage manufacturers, civil society groups, and public health advocates. The consequent delay resulted in the HPL being signed into law in December, 2017, and formally implemented on April 1, 2018. This process saw substantial concessions made to both the sugar and beverage industries, with the effective tax burden being reduced from 20% to approximately 10–11%. The particular rate of the HPL depends on the sugar content of the beverage, at 0·021 ZAR per gram of sugar, above a threshold of 4 g of sugar per 100 mL.^26^ Small producers of taxable beverages using less than 500 kg of sugar per year are exempt from paying the HPL.

There is extensive literature simulating SSB tax policies in the South African context, and exploring related issues,^27-31^ but the literature on the observed impacts of the HPL is only beginning to emerge, given its recent implementation. A recent study documenting changes in SSB prices following the HPL implementation found pre-VAT price increases averaging 1·006 ZAR per litre for carbonates (an approximate 6% increase), and no increase for non-taxable products such as bottled water and 100% fruit juices.^32^ In this Article, we assess changes in household purchases of taxable and non-taxable beverages in terms of volume, sugar, and calories following the HPL’s introduction. Given the lengthy process from the HPL’s announcement to its implementation, we assess changes after the announcement period (March, 2016, onwards) and the implementation period (April, 2018, onwards) compared with the pre-announcement period. We also assess differential changes in purchasing behaviours of households stratified by household socioeconomic status.

## Methods

### Data

This study uses data on household purchases collected by Kantar Europanel in South Africa. The sample contains approximately 3000 households each year reporting household purchases, from January, 2014, to March, 2019 (n=113 653 household-month observations in total). Panel members are instructed to record all items purchased on all household members’ shopping trips, including the item’s barcode using scanners provided to them along with a barcode booklet for products without barcodes (eg, cut-to-order meat or unpackaged fruit and vegetables). Panel members are also instructed to provide information about where they shopped and date of the shopping episode, and submit photos of their receipts via the data collection system. The sample of households includes urban and rural households. However, due to the potential absence of electricity required for recording purchases, the sample excludes extremely poor households, classified as Living Standards Measure (LSM) 1–3, which represent approximately 5–10% of the South African population (appendix p 2). LSM is a widely used marketing research tool developed by the South African Audience Research Foundation and is derived from a set of questions (29 in the 2011 update) around household ownership of goods; access to water, electricity, media, and financial services; area and type of residence; education; and income. Households are recruited via telephone, text, and online, with poor reporters (ie, those who do not report more than five different categories of items purchased or have fewer than one shopping trip per week) being dropped on a rolling basis with immediate targeted replenishment based on sociodemographic attributes. These strict criteria and the replenishment approach meant that 38% of households in the sample had no more than six (monthly) observations, 54% of households no more than 12 observations, and 72% of households no more than 24 observations.

Along with the survey weights, the sample is meant to represent 13·7 million households, or 42–45 million people from all nine provinces, covering approximately 90–95% of the country’s population. To allow for comparison with national demographic measures, we present the sample’s survey-weighted sociodemographic characteristics for each year of data included in this analysis (table 1). When compared with the sociodemographic characteristics of the Statistics South Africa’s General Household Survey (appendix p 3), we find the age of household head is slightly younger but otherwise the weighted Kantar sample is broadly similar. Since we only have household purchases from January to March in 2019, the number of unique households for 2019 is smaller.

To identify the nutritional content of household beverage purchases, we merged the Kantar household purchase data with nutrition panel data obtained from several sources. This included the Mintel Global New Product Database, with data from 2015 to 2019; nutrition panel data collected by Discovery Vitality and The George Institute in 2015, 2016, and 2017; and nutrition panel data collected by our research team in 2018 and 2019 following a standardised protocol for data collection and entry and reviews by our research team to exclude implausible or inconsistent nutrient values. The household purchase data and nutrition panel data were then matched by barcode and time period to maximise accuracy in how beverages purchased were categorised as taxable or non-taxable based on the South Africa Revenue Service regulations on tariff codes. Since tariff codes are broad for some beverage categories (eg, carbonated beverages), there are beverages under taxable tariff codes that have tax rates of 0 ZAR (eg, carbonated beverages with sugar content <4 g/100 mL); we consider such products as taxable in this analysis. The various beverage categories included in this study, along with their HPL taxable versus non-taxable status and corresponding tariff codes are shown in the appendix (p 4).

This study received exemption from requiring institutional review board approval since all data were de-identified and secondary in nature.

### Outcomes

We constructed three outcome measures of beverage purchases: sugar content expressed in g/capita per day, calories in kcal/capita per day (since some beverages also contain fat, protein, and other forms of carbohydrates), and volume in mL/capita per day. These outcome measures were aggregated across classes of beverage categories based on being HPL taxable or non-taxable.

### Statistical analysis

To quantify the changes in purchasing patterns following the introduction of the HPL, we first constructed simple survey-weighted means of the beverage purchase outcomes across three salient periods: pre-announcement of the intent to legislate (January, 2014, to February, 2016), post announcement but pre-implementation (March, 2016, to March, 2018), and post implementation (April, 2018, to March, 2019). In addition, we stratified our sample by LSM, reporting means for lower LSM households (LSM 4–6) as well as higher LSM households (LSM 7–10).

To assess changes in households’ purchases of beverages compared with what they might have been had the HPL not been announced or implemented, we fitted household fixed-effects regression models that controlled for household factors (household size, number of adults, lifecycle stage [ie, young without children; families; so-called empty nesters, whose children have left home; mature without children; and retired], and total food and non-food spending) and subnational time-varying factors, specifically consumer price indices, that could be driving changes in the outcomes across the three policy periods. The model allows for level shifts across the policy periods and simultaneously allows for period-specific trends. Using the models’ implied pre-HPL trends, we forecast naive counterfactuals into the post-announcement and post-implementation periods. We then compared predicted adjusted outcomes with the pre-trend prediction counterfactual outcomes to estimate both absolute and relative differences in sugar, calories, and volumes. We estimated models for the full sample, as well as stratified by lower LSM and higher LSM households.

To aid interpretation of the findings, we report results in terms of mean predicted outcomes rather than regression coefficients, with 95% CIs constructed from bootstrapped SEs. Further technical detail of our methodological approach is provided in the appendix (p 1), covering model specifications for the trend analyses, the prediction approach, and how we derived the bootstrapped SEs. All analyses were done in Stata (version 16).

### Role of the funding source

Funders of the study had no role in study design, data collection, data analysis, data interpretation, or writing of the report.

## Results

The unadjusted survey-weighted means of daily sugar, calories, and volume indicate that, without accounting for other potential factors, there were reductions in sugar, calories, and volume for taxable beverages in the full sample in the post-announcement and the post-implementation periods compared with the pre-announcement period (table 2). We found sugar from taxable beverages fell from 16·25 g/capita per day (95% CI 15·80–16·70) in the pre-announcement period to 10·63 (10·22–11·04) in the post-implementation period and the volume of beverage purchases fell from 518·99 mL/capita per day (506·90–531·08) to 443·39 (430·10–456·56). Overall, there were small changes in the purchase outcomes for non-taxable beverages (table 2).

Lower LSM households bought more taxable beverages than did higher LSM households in the pre-announcement period, but experienced larger reductions in purchases in the post-announcement and post-implementation periods (table 2). Lower LSM households purchased on average 8·2 fewer g/capita per day of sugar from SSBs over the first year of the HPL being in place compared with the pre-announcement period. By contrast, the mean reduction in sugars from SSBs for higher LSM households was 2·1 g/capita per day.

The unadjusted survey-weighted mean amounts of sugar, calories, and volume per capita, by beverage type, during each of the three periods are shown in the appendix (p 5). Taxable carbonates accounted for most of the taxable beverages and drove the reductions in sugar, calories, and volume. Among non-taxable beverages, there was a slight increase in sugar, calories, and volume from 100% fruit juices (appendix p 5).

Regression coefficients for the full sample models for each of the outcomes of interest are shown in the appendix (p 6). These results were used to predict the naive counterfactual as the basis for comparing the regression-adjusted outcomes in the post-announcement and post-implementation policy periods.

**Figure 1:**
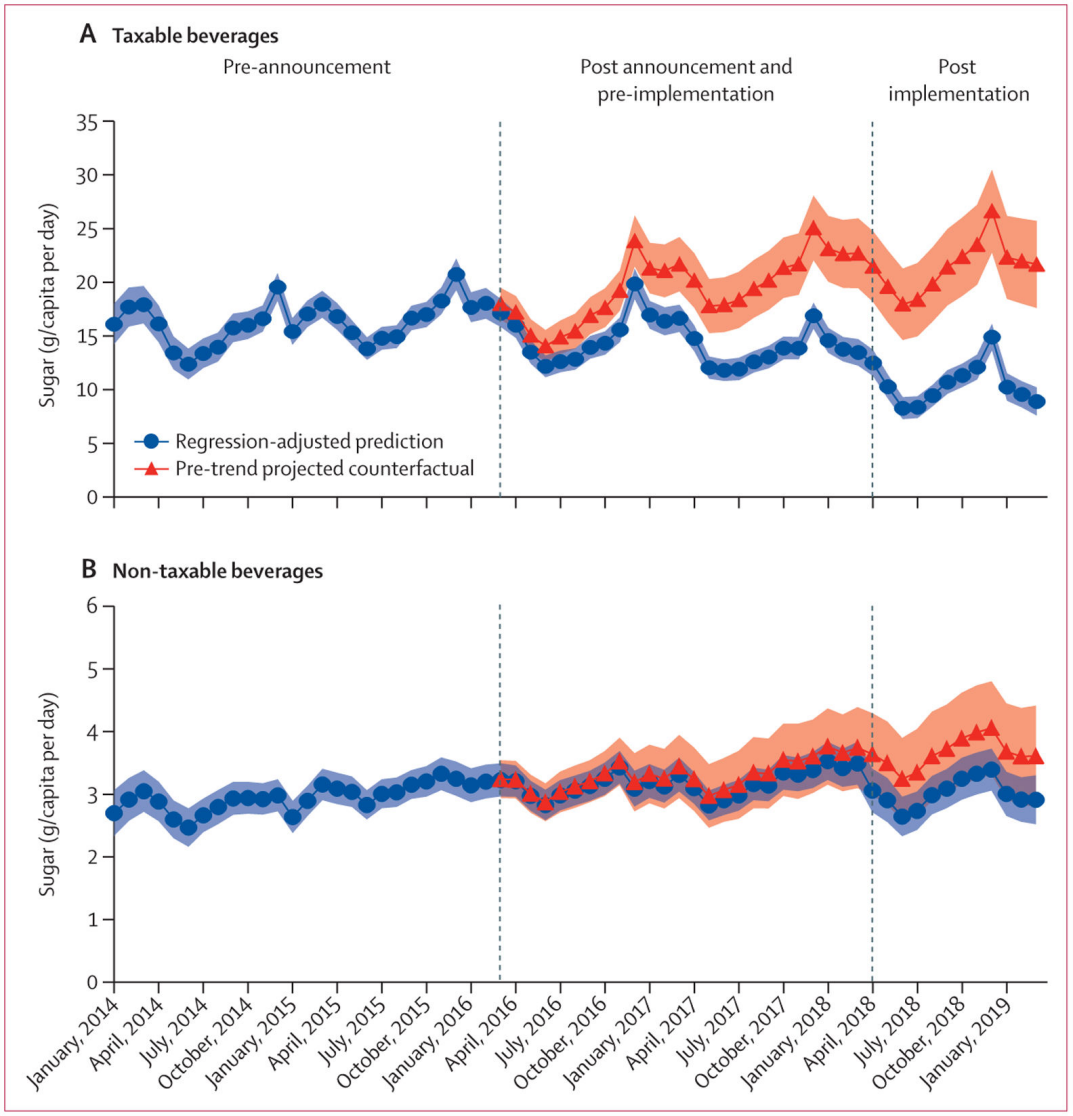
Sugar from beverage purchases (g/capita per day) Regression-adjusted predicted values are compared with pre-trend counterfactuals, projected using the pre-announcement regression values. Mean values represent 13·7 million South African households with LSM of at least 4. Data covers household purchases from January, 2014, to March, 2019. Shaded regions are 95% CIs. LSM=Living Standards Measure.

**Figure 2:**
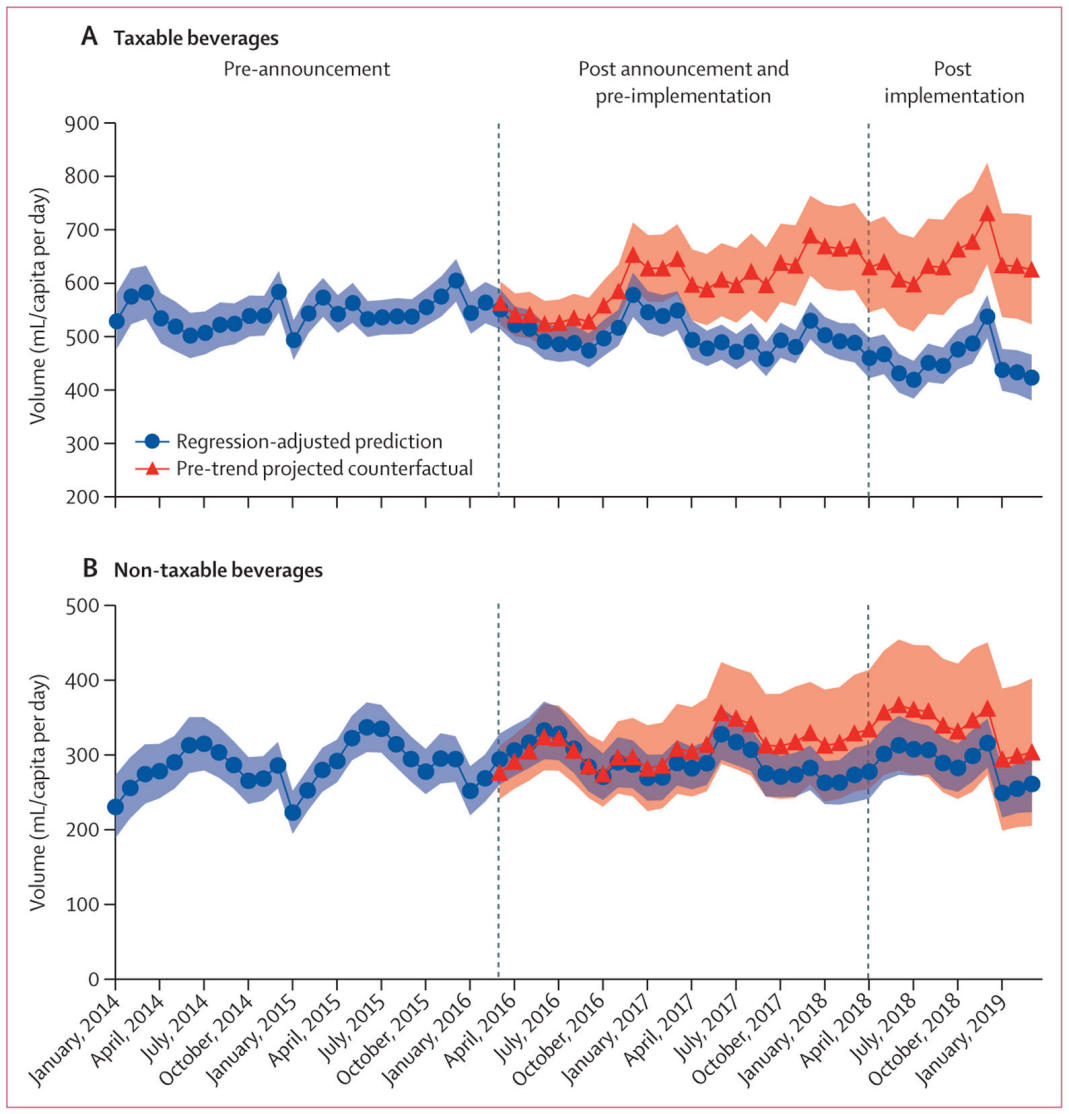
Volume from beverage purchases Regression-adjusted predicted values are compared with pre-trend counterfactuals, projected using the pre-announcement regression values. Mean values represent 13·7 million South African households with LSM of at least 4. Data covers household purchases from January, 2014, to March, 2019. Shaded regions are 95% CIs. LSM=living standards measure.

Our predicted values for sugar, calories and volume from taxable beverages from the regression analyses showed that there were already large reductions in sugar purchased from taxable beverages in the post-announcement period (figure 1A), representing a reduction of 26% compared with the pre-trend projected counterfactual (table 3). Reductions in calories were commensurate, with a reduction of 27%, and volume of taxable beverages purchased also fell (figure 2A), representing a 16% reduction. These reductions grew over the first year of the HPL (post implementation; figure 1), with sugar purchased from taxable beverages being 51% lower compared with the counterfactual based on the pre-announcement trends (table 3). Likewise, both relative and absolute differences in calories from and volume of taxable beverages were also larger in the post-implementation period compared with the post-announcement period (table 3; figure 2A).

Importantly, consistent with the descriptive findings, in both the post-announcement and post-implementation periods, the significant reductions for taxable beverages compared with the counterfactual were consistently larger among lower LSM households in both absolute and relative terms compared with higher LSM households (table 4).

For non-taxable beverages, we found non-significant differences in purchases between the counterfactual and the regression-adjusted outcomes during both post-announcement and post-implementation periods (table 3; figures 1B, 2B). Results for non-taxable beverages by LSM are shown in the appendix (p 7). Again, we found non-significant differences, except for calories post implementation, where lower LSM households consumed fewer calories from non-taxable beverages (appendix p 7).

## Discussion

General guidance from WHO recommends taxation on the basis of sugar content of SSBs, but there is little empirical evidence on the effects of such taxes.^25^ To our knowledge, this study is the first assessment of changes in South African households’ purchases of taxable and non-taxable beverages before and after the sugar content-based HPL was announced and implemented. We found mean sugars and volume of taxed beverage purchases fell, while no changes were observed for non-taxable beverages. Moreover, when we compare regression-adjusted predictions to counterfactual scenarios where the announcement and implementation did not occur, taxable purchases were considerably lower. While we cannot establish causality, we can make some inferences. Taken together these findings suggest that the HPL contributed to lower SSB purchases and, possibly, a reduction in the intake of sugars, calories, and volume from taxable SSBs.

Our findings also align with economic theory and empirical evidence, including earlier findings in Mexico,^13,33^ showing larger relative reductions in purchases of taxable beverages among lower LSM households compared with reductions observed in higher LSM households. Because lower LSM households in South Africa purchased considerably higher amounts (by nearly 230 mL/capita per day) of SSBs before the HPL announcement, a larger relative and absolute reduction among this subpopulation means that the HPL might be progressive for health. This is because of the greater price sensitivity, interacting with brands’ sugar reformulation, and the greater burden of poor health (loss of earnings and human capital) in lower-income households and individuals.^33,34^ We should note, however, that our sample did not include the lowest LSM households, who make up 5–10% of the population.

Compared with evaluations of volume-based SSB excise taxes to date, the reductions here are considerably larger. For example, in Mexico, the 9% price increase was associated with a 6% reduction in volume of SSBs purchased compared with counterfactuals over the first year.^13^ In South Africa, we found the sugar-based HPL resulted in a 16% reduction in volume purchased after the HPL announcement and a 28% reduction after its implementation, compared with similar counterfactuals. The UK adopted a tiered but also sugar-based design, and a recent study found that between 2015 and 2018, the volume of sales of soft drinks that are subject to the tax fell by 50%, while volume sales of those not subject to the tax increased by 40%.^35^ This was equivalent to a net reduction of 4·6 g/capita.^35^ However, as there are substantial differences across these countries, including in underlying incomes and levels of beverage purchases, direct inferences from this literature to the effectiveness of alternate designs might not be possible. Further research is needed to understand to what extent the designs of these policies are driving the observed differences in outcomes observed across settings.

Changes in purchases in South Africa began after the announcement of the intention to pursue an SSB tax policy, suggesting that consumption is driven by not only consumers’ response to greater awareness of the harms of SSBs as part of the discussions around the HPL, but also by anticipatory response from the beverage industry. The announcement in June, 2016, from the National Treasury signalled an intention to levy a sugar-based tax.^24^ This seems to have triggered anticipatory sugar-content reduction by volume and other strategies such as downsizing of packages^36^ in the run-up to, as well as after, the implementation of the tax policy. It is also possible that there were demand-side responses, related to high levels of press coverage of the policy debates and media campaigns. These could have increased salience of the health concerns surrounding SSB consumption before tax implementation. Moreover, the subsequent HPL implementation is expected to have induced demand changes. Based on the descriptive before-and-after volume changes between the post-announcement and post-implementation periods, there was a 9·9% reduction across all households in this study (15·6% reduction among lower LSM households).

Regardless, the larger relative reduction in sugars compared with volume is evidence that there were changes in both quantities purchased by households and the sugar content of taxable beverages. The scope of this study was restricted to quantifying the magnitude of observed changes in purchases; however, future work could more fully interrogate the mechanisms underlying the changes documented here and the extent of reformulation. Several countries are implementing other food policies such as front-of-packet labelling or marketing restrictions based on the levels of nutrients such as sugar, sodium, and saturated fats.^37^ In response to these mandatory regulations and legislation, food and beverage manufacturers are likely to be developing new food technologies to partially lower these nutrients in their products. However, what remains unknown are the long-term health implications of sugar and fat substitutes, especially as their use increases. Future food-related and beverage-related policies in South Africa should consider monitoring the use and amount of these additives in beverages and foods.

This study has some key limitations. First, the Kantar data did not cover the lowest LSM subset of the South African population (LSM 1–3), which represented around 5–10% of the country’s population. Other ongoing studies are seeking to fill this gap and, together with this current analysis, can provide a richer picture of how the HPL is associated with changing behaviours of both consumers and the beverage industry. However, our findings regarding differences in purchase changes by socioeconomic status are consistent with those of Mexico and elsewhere.^13^ Second, since the HPL is a national policy, there is no true control and thus causality of this policy cannot be inferred. To address this, we adopted an approach that has been used widely in other national SSB tax evaluations by forecasting purchase outcomes based on pre-HPL trends as comparison.^13,23,38^ As noted above, our findings of the tax affecting beverage purchases are consistent with this literature.

Key strengths of this work include the use of detailed household purchase data spanning 63 months, carefully linked in a temporal manner to nutrition label data at the barcode level. We also consulted with lawyers and verified with the National Treasury to ensure that our interpretations of what items were taxable were consistently applied.

In conclusion, the announcement and implementation of South Africa’s sugar content-based SSB tax, the HPL, has coincided with large reductions in purchases in terms of volumes and sugar quantities from taxable beverages, with non-significant changes for non-taxable beverages. While other countries in sub-Saharan Africa have levied SSB taxes, this is the first country in the region to evaluate such a policy, and our results clearly show positive changes that could offer useful public health gains across the region. The reductions in sugar from taxable beverage purchases suggest a potential role for sugar-based taxes more broadly.

## Supplementary Material

1

## Figures and Tables

**Table 1:** Survey-weighted Kantar household sample characteristics

	2014 (n=3073)	2015 (n=3135)	2016 (n=2902)	2017 (n=3012)	2018 (n=2780)	2019 (n=2117*)
**Demographics**						
Age of household head	37·9 (37·3–38·4)	39·0 (38·4–39·6)	38·3 (37·6–39·0)	38·6 (37·8–39·3)	39·1 (38·4–39·8)	40·2 (39·4–41·1)
Number of adults	2·4 (2·3–2·5)	2·3 (2·3–2·4)	2·5 (2·4–2·6)	2·5 (2·4–2·6)	2·4 (2·4–2·5)	2·5 (2·4–2·5)
Number of children	1·0 (1·0–1·1)	0·9 (0·8–0·9)	0·8 (0·7–0·9)	1·0 (0·9–1·0)	1·0 (0·9–1·0)	0·9 (0·8–1·0)
Lifecycle stage						
Young without children	20% (18–22)	18% (16–20)	24% (22–27)	20% (17–22)	17% (15–19)	14% (12–17)
Families	56% (53–58)	51% (49–54)	46% (44–49)	53% (50–56)	55% (52–57)	53% (50–56)
Empty nesters†	8% (7–9)	11% (10–13)	11% (9–12)	10% (9–12)	11% (9–12)	12% (10–14)
Mature without children	15% (14–17)	18% (16–20)	17% (15–19)	15% (13–17)	15% (13–17)	17% (15–19)
Retired	1% (1–2)	2% (1–3)	2% (2–3)	3% (2–3)	3% (3–4)	4% (3–5)
**LSM**						
LSM 4	9% (8–11)	8% (7–10)	8% (6–10)	8% (6–10)	10% (8–12)	10% (8–13)
LSM 5	12% (10–13)	12% (10–14)	14% (12–17)	16% (14–18)	17% (15–20)	17% (14–20)
LSM 6	33% (30–35)	38% (35–40)	38% (35–41)	35% (33–38)	33% (31–36)	33% (30–36)
LSM 7	14% (12–15)	14% (12–15)	13% (11–15)	13% (12–15)	13% (12–15)	12% (11–14)
LSM 8	12% (11–14)	12% (10–13)	11% (9–12)	10% (9–12)	11% (9–12)	11% (9–12)
LSM 9	14% (12–15)	12% (10–13)	11% (10–13)	12% (10–13)	11% (9–12)	12% (10–14)
LSM 10	8% (7–9)	5% (5–6)	6% (5–7)	6% (5–7)	5% (4–6)	5% (4–6)
**Province**						
Western Cape	15% (13–17)	11% (10–13)	12% (10–14)	13% (11–15)	12% (10–14)	12% (10–14)
Eastern Cape	9% (8–11)	10% (9–13)	9% (7–12)	8% (6–10)	9% (7–12)	7% (5–9)
Northern Cape	2% (1–3)	1% (1–3)	1% (0–2)	1% (0–2)	0% (0–1)	0% (0–1)
Free State	7% (6–8)	7% (6–8)	8% (6–9)	7% (6–9)	8% (7–10)	9% (7–11)
KwaZulu-Natal	15% (13–17)	15% (13–17)	16% (14–19)	19% (17–21)	16% (14–18)	167% (14–19)
North West	6% (5–8)	6% (5–8)	7% (6–9)	6% (4–7)	6% (5–7)	5% (4–7)
Gauteng	31% (29–33)	33% (31–35)	30% (28–32)	29% (27–32)	30% (28–33)	32% (29–34)
Mpumalanga	5% (4–6)	6% (5–7)	5% (4–7)	6% (5–8)	8% (6–10)	7% (5–9)
Limpopo	11% (9–13)	10% (9–12)	12% (10–15)	12% (10–15)	11% (10–14)	12% (10–15)

Data are mean (95% CI) or % (95% CI). Mean values are survey weighted to be representative of around 13·7 million South African households with LSM of 4 or higher. LSM=Living Standards Measure.

* Number of unique households was smaller in 2019 because this only included data from January to March.

† Parents whose children have left home.

**Table 2:** Unadjusted survey-weighted mean beverage purchases, overall and by LSM

	Full sample		Lower LSM (4–6)		Higher LSM (7–10)	
	Taxable beverages	Non-taxable beverages	Taxable beverages	Non-taxable beverages	Taxable beverages	Non-taxable beverages
**Daily sugar (g/capita per day)**						
Pre-announcement	16·25 (15·80–16·70)	2·72 (2·64–2·80)	18·63 (17·90–19·36)*	2·39 (2·29–2·49)*	13·10 (12·69–13·51)	3·15 (3·03–3·27)
Pre-implementation, post announcement	14·26 (13·85–14·67)†	3·05 (2·97–3·13)†	15·43 (14·78–16·08)†*	2·51 (2·41–2·61)*	12·56 (12·15–12·97)†	3·83 (3·69–3·97)†
Post implementation	10·63 (10·22–11·04)†‡	3·09 (2·97–3·21)	10·39 (9·88–10·90)†‡	2·37 (2·23–2·51)*	10·97 (10·31–11·61) †‡	4·11 (3·87–4·35)†
**Daily calories (kcal/capita per day)**						
Pre-announcement	70·21 (68·31–72·11)	45·14 (43·67–46·61)	80·27 (77·19–83·35)*	43·65 (41·45–45·85)	56·91 (55·15–58·67)	47·1 (45·34–48·86)
Pre-implementation, post announcement	62·45 (60·67–64·23)†	49·25 (47·68–50·82)†	67·24 (64·50–69·98)†*	46·46 (44·15–48·77)*	55·45 (53·69–57·21)	53·34 (51·48–55·20)†
Post implementation	46·45 (44·71–48·15)†‡	45·12 (43·24–46·96)‡	45·22 (43·02–47·42)†‡	37·57 (35·16–39·94)‡*	48·21 (45·41–50·93)†‡	55·87 (52·93–58·77)†
**Daily volume (mL/capita per day)**						
Pre-announcement	518·99 (506·90–531·08)	283·45 (273·34–293·56)	616·04 (596·07–636·01)*	261·83 (246·17–277·49)*	390·70 (381·00–400·40)	312·02 (301·02–323·02)
Pre-implementation, post announcement	492·16 (481·28–503·04)†	310·53 (298·34–322·72)†	555·49 (538·79–572·19)†*	284·98 (266·36–303·60)*	399·45 (388·57–410·33)	347·94 (335·44–360·44)†
Post implementation	443·39 (430·10–456·56)†‡	312·94 (296·29–329·29)†	468·89 (449·66–488·12)†‡*	263·36 (243·70–282·78)*	407·08 (390·18–423·66)	383·53 (354·95–411·79)†

Data are mean (95% CI). Mean values are survey weighted to be representative of 13·7 million South African households with LSM of 4 or higher, and cover household purchases from January, 2014, to March, 2019. LSM=Living Standards Measure.

* Significant difference from higher LSM values at p<0·01.

† Significant difference from pre-announcement values at p<0·01.

‡ Significant difference from pre-implementation and post-announcement values at p<0·01.

**Table 3:** Regression-predicted taxable and non-taxable beverage purchases

	Predicted purchases	Pre-trend counterfactual purchases	Absolute difference	Relative difference
**Taxable beverages**				
Pre-implementation, post announcement				
Sugar (g/capita per day)	14·35 (13·49 to 15·21)	19·42 (17·25 to 21·59)	−5·07 (−6·90 to −3·25)	−26·1%
Calories (kcal/capita per day)	62·60 (58·83 to 66·37)	85·79 (76·57 to 95·00)	−23·19 (−30·82 to −15·56)	−27·0%
Volume (mL/capita per day)	503·28 (476·93 to 529·62)	599·81 (544·65 to 654·98)	−96·53 (−141·73 to −51·34)	−16·0%
Post implementation				
Sugar (g/capita per day)	10·53 (9·58 to 11·48)	21·39 (17·91 to 24·88)	−10·86 (−14·21 to −7·51)	−50·8%
Calories (kcal/capita per day)	45·77 (41·66 to 49·88)	95·81 (81·14 to 110·48)	−50·04 (−64·07 to −36·00)	−52·2%
Volume (mL/capita per day)	455·15 (423·97 to 486·33)	640·40 (552·59 to 728·20)	−185·24 (−269·42 to −101·07)	−28·9%
**Non-taxable beverages**				
Pre-implementation, post announcement				
Sugar (g/capita per day)	3·16 (2·97 to 3·36)	3·30 (2·88 to 3·72)	−0·13 (−0·49 to 0·22)	−3·9%
Calories (kcal/capita per day)	50·96 (46·56 to 55·36)	53·17 (44·92 to 61·41)	−2·21 (−8·65 to 4·24)	−4·3%
Volume (mL/capita per day)	292·15 (268·27 to 316·03)	311·25 (258·64 to 363·85)	−19·10 (−65·80 to 27·61)	−5·1%
Post implementation				
Sugar (g/capita per day)	3·01 (2·71 to 3·30)	3·64 (2·96 to 4·33)	−0·64 (−1·29 to 0·02)	−17·6%
Calories (kcal/capita per day)	45·08 (40·76 to 49·39)	59·67 (46·95 to 72·40)	−14·6 (−26·80 to −2·40)	−17·3%
Volume (mL/capita per day)	289·05 (260·57 to 317·53)	338·31 (251·47 to 425·15)	−49·26 (−132·67 to 34·15)	−14·6%

Data are mean (95% CI). Values represent 13·7 million South African households with LSM of 4 or higher. Regressions are adjusted for household characteristics (household size, number of adults, lifecycle stage, and total food and non-food spending) and provincial characteristics (consumer price indices). LSM=Living Standards Measure.

**Table 4:** Regression-predicted taxable beverage purchases by LSM

	Predicted purchases	Pre-trend counterfactual purchases	Absolute difference	Relative difference
**Lower LSM (4–6)**				
Pre-implementation, post announcement				
Sugar (g/capita per day)	15·65 (14·37 to 16·94)	22·63 (19·07 to 26·18)	−6·97 (−9·93 to −4·02)	−30·8%
Calories (kcal/capita per day)	68·34 (62·75 to 73·93)	100·12 (84·82 to 115·42)	−31·78 (−44·31 to −19·25)	−31·7%
Volume (mL/capita per day)	585·44 (545·80 to 625·08)	704·41 (609·07 to 799·75)	−118·97 (−203·53 to −34·40)	−16·9%
Post implementation				
Sugar (g/capita per day)	10·54 (9·11 to 11·96)	24·56 (18·72 to 30·41)	−14·03 (−19·52 to −8·54)	−57·1%
Calories (kcal/capita per day)	45·38 (39·25 to 51·50)	109·57 (84·49 to 134·65)	−64·20 (−87·62 to −40·77)	−58·6%
Volume (mL/capita per day)	495·57 (446·93 to 544·21)	724·62 (565·99 to 883·24)	−229·05 (−377·71 to −80·39)	−31·6%
**Higher LSM (7–10)**				
Pre-implementation, post announcement				
Sugar (g/capita per day)	13·36 (12·32 to 14·39)	17·11 (14·75 to 19·46)	−3·75 (−5·85 to −1·64)	−21·9%
Calories (kcal/capita per day)	59·63 (55·12 to 64·15)	76·75 (66·80 to 86·70)	−17·12 (−25·95 to −8·28)	−22·3%
Volume (mL/capita per day)	441·20 (413·73 to 468·68)	525·48 (464·81 to 586·15)	−84·28 (−134·13 to −34·43)	−16·0%
Post implementation				
Sugar (g/capita per day)	10·63 (9·39 to 11·88)	19·14 (15·38 to 22·90)	−8·51 (−12·28 to −4·73)	−44·5%
Calories (kcal/capita per day)	48·03 (42·40 to 53·67)	87·43 (71·52 to 103·34)	−39·40 (−55·17 to −23·62)	−45·1%
Volume (mL/capita per day)	427·95 (389·04 to 466·86)	584·34 (485·70 to 682·99)	−156·40 (−251·37 to −61·42)	−26·8%

Data are mean (95% CI). Values represent 13·7 million South African households with LSM of 4 or higher. Regressions are adjusted for household characteristics (household size, number of adults, lifecycle stage, total food and non-food spending) and provincial characteristics (consumer price indices). LSM=Living Standards Measure.

## Research in context

### Evidence before this study

Sugar-sweetened beverage (SSB) taxes are gaining popularity as a policy option for reducing excessive sugar intake in more and more countries. A 2019 systematic review and meta-analysis showed that taxes have contributed to lowering the volume of SSBs purchased. In 2018, South Africa introduced a sugar-based tax on SSBs known as the Health Promotion Levy (HPL). Although there is existing work simulating SSB tax policies in the South African context, the literature on the observed impacts of the HPL has not yet been peer reviewed and published.

### Added value of this study

As the first African nation to implement a sugar content-based tax, together with its influence in the region and on the continent, the South African context is a useful addition to the body of evidence on SSB taxes. South Africa’s HPL has a unique design based on sugar content, with each gram of sugar beyond 4 g (per 100 mL of beverage) being taxed. This analysis also provides an assessment of purchase differentials between households by socioeconomic status.

### Implications of all the available evidence

SSB taxes levied according to sugar content can help to reduce excessive sugar and energy intake. Future work should be done to understand the longer-term health implications for South Africans.
