# Supplementary material for: Changes in beverage purchases following the announcement and implementation of South Africa’s Health Promotion Levy: an observational study
Source: Lancet Planet Health. Author manuscript; Available in PMC 2021 Apr 25. (PMC8071067; doi:10.1016/S2542-5196(20)30304-1)
Supplement: 1 [file NIHMS1692779-supplement-1.pdf]

THE LANCET
Planetary Health

Supplementary appendix

This appendix formed part of the original submission and has been peer reviewed. We post it as supplied by the authors.

Supplement to: Stacey N, Edoka I, Hofman K, Swart EC, Barry Popkin, Ng SW. Changes in beverage purchases following the announcement and implementation of South Africa's Health Promotion Levy: an observational study. *Lancet Planet Health* 2021; **5:** e200–08.

SUPPLEMENTARY MATERIALS

Methodological Detail 1

Supplemental Table S1: StatsSA General Household Survey, Survey-Weighted Population by LSM, 2014 and 2018 2

Supplemental Table S2: StatsSA General Household Survey, LSM 4-10 only Sample Characteristics, 2014 - 2018 3

Supplemental Table S3: Beverage types by tax status and tariff codes 4

Supplemental Table S4: Survey weighted purchases by beverage types 5

Supplemental Table S5: Regression Coefficients 6

Supplemental Table S6: Non-Taxable Beverage Purchases Relative to Pre-Trend by LSM 7

References 8

Methodological Detail

While the simple means presented in Table 2 are informative about changes in beverage purchases across the three periods of interest, the changes they demonstrate could be a product of other contemporaneous changes. We thus use regressions to adjust for other co-varying factors that could explain the variation in purchase outcomes. In particular we include controls for household characteristics (household size, number of adults, life-cycle stage, total food and non-food spending) and provincial consumer price index measures to adjust for non-tax related determinants of beverage purchases that could confound estimates of the changes beverage purchase trends.

Exploiting repeated observations among households in the data, we run household-fixed effects regression models of the following form:

$$y_{ht} = \beta_0 + \beta_1 \text{Pre}_t \times \text{Time}_t + \beta_2 \text{PostAnnouncement}_t + \beta_3 \text{PostAnnouncement}_t \times \text{Time}_t \\ + \beta_3 \text{PostImplementation}_t + \beta_4 \text{PostImplementation}_\text{t} \times \text{Time}_\text{t} + \boldsymbol{\beta}_5' \mathbf{x}_{\boldsymbol{ht}} + \nu_h + \epsilon_{ht}$$

where h indexes households, t indexes time period, y_{ht} is the particular outcome measure, $\mathbf{x}_{\boldsymbol{ht}}$ is a vector of time-varying household characteristics including household demographics, ν_h is a household fixed-effect, and ϵ_{ht} is a mean zero, idiosyncratic error term. The time-defined terms: $\beta_1 \text{Pre}_t \times \text{Time}_t + \beta_2 \text{PostAnnouncement}_t + \beta_3 \text{PostAnnouncement}_t \times \text{Time}_t + \beta_3 \text{PostImplementation}_t + \beta_4 \text{PostImplementation}_t \times \text{Time}_t$, constitute a piece-wise linear function in time. This allows for level shifts across of the policy periods, and simultaneously allows for period-specific trends. We fit this model for three outcomes: beverage sugar per capita per day, beverages calories per capita per day, and volume per capita per day; and for three sub-population: full sample, lower LSM (LSM 4-6), higher LSM (LSM 7-10). Estimated coefficients are reported in Supplemental Table S3. All models were fitted in STATA version 16.

To aid interpretation of the findings, we report results in terms of mean predicted outcomes (i.e. $\overline{\hat{y}_{ht}} = \frac{1}{N_{ht}} \cdot \sum_{ht} \hat{y}_{ht}$) rather than regression coefficients. We construct standard errors for these mean predictions by bootstrap (1000 draws of bootstrap sample of 6000 households). We compare mean predicted value to a counterfactual constructed by projecting the pre-trend into the post-announcement and post-implementation periods. These results are reported on a policy period basis in Table 3 and Table 4, and on a monthly basis in Figure 1 and Figure 2. The approach is broadly consistent with prior approaches to assessing changes in purchasing behaviors following the introduction of national sugar-sweetened beverage taxes (1-4). Rather than specifying the exact functional form ex-ante, we adopted multiple generic polynomial forms for the time trends. We used AIC and BIC as measures of model goodness-of-fit and found that across the outcomes, these were very similar for linear time trends vs higher-order time trends. Consequently, for ease in interpretation and consistency across multiple outcomes, we present our results based on linear time-trends.

Supplemental Table S1: StatsSA General Household Survey, Survey-Weighted Population by LSM, 2014 and 2018

		2014			2018		
		Proportion of Households	Population (000s)	Total Population (000s) **[%]**	Proportion of Households	Population (000s)	Total Population (000s) **[%]**
Living Standards Measure:							
Population excluded from Kantar sample	1	0.01<br>(0.01, 0.01)	300<br>(228, 372)	4,876<br>(4,494, 5,258)<br>**[9.12%]**	0.00<br>(0.00, 0.00)	55<br>(24, 85)	1,804<br>(1,619, 1,990)<br>**[3.35%]**
	2	0.03<br>(0.03, 0.03)	1,312<br>(1,162, 1,463)		0.01<br>(0.01, 0.01)	315<br>(235, 394)	
	3	0.08<br>(0.07, 0.08)	3,264<br>(2,958, 3,570)		0.03<br>(0.03, 0.04)	1,435<br>(1,291, 1,580)	
Population included in Kantar sample	4	0.15<br>(0.14, 0.16)	6,796<br>(6,428, 7,164)	48,572<br>(47,384, 49,761)<br>**[90.88%]**	0.13<br>(0.13, 0.14)	5,597<br>(5,312, 5,883)	52,122<br>(51,303, 52,940)<br>**[96.65%]**
	5	0.22<br>(0.21, 0.23)	12,257<br>(11,722, 12,792)		0.23<br>(0.22, 0.23)	11,680<br>(11,257, 12,104)	
	6	0.24<br>(0.23, 0.25)	14,810<br>(14,179, 15,440)		0.29<br>(0.29, 0.30)	17,702<br>(17,155, 18,248)	
	7	0.09<br>(0.08, 0.09)	5,034<br>(4,709, 5,359)		0.11<br>(0.11, 0.12)	6,605<br>(6,275, 6,934)	
	8	0.06<br>(0.05, 0.06)	3,222<br>(2,930, 3514)		0.07<br>(0.06, 0.07)	3,878<br>(3,626, 4,131)	
	9	0.07<br>(0.07, 0.08)	3566<br>(3183, 3,950)		0.07<br>(0.07, 0.08)	3,967<br>(3,697, 4,237)	
	10	0.06<br>(0.05, 0.06)	2,888<br>(2,599, 3,176)		0.05<br>(0.05, 0.05)	2,693<br>(2,450, 2,936)	

Notes: Calculated from Statistics South Africa General Household Survey, 2014 and 2018. Excluded population are households in LSMs 1-3, whom are excluded from the Kantar Europanel used for the primary analyses of this paper. Included population are households in LSMs 4-10 who are included in the Kantar Europanel used for the primary analyses of this paper.

Supplemental Table S2: StatsSA General Household Survey, LSM 4-10 only Sample Characteristics, 2014 - 2018

	2014	2015	2016	2017	2018
Demographics: (mean)					
Age of household head	46.370	46.452	46.311	45.657	45.748
	(46.04 - 46.70)	(46.18 - 46.72)	(46.05 - 46.57)	(45.39 - 45.92)	(45.48 - 46.02)
Number of adults	2.273	2.224	2.173	2.137	2.136
	(2.250 - 2.295)	(2.204 - 2.245)	(2.153 - 2.192)	(2.118 - 2.156)	(2.117 - 2.155)
Number of children	1.235	1.190	1.168	1.145	1.130
	(1.207 - 1.263)	(1.167 - 1.214)	(1.144 - 1.191)	(1.122 - 1.169)	(1.107 - 1.153)
Living Standards Measure: (Proportion)					
LSM 4	0.169	0.169	0.147	0.138	0.137
	(0.161 - 0.178)	(0.163 - 0.176)	(0.141 - 0.154)	(0.132 - 0.144)	(0.132 - 0.144)
LSM 5	0.250	0.257	0.244	0.243	0.236
	(0.242 - 0.259)	(0.250 - 0.264)	(0.238 - 0.251)	(0.236 - 0.250)	(0.229 - 0.243)
LSM 6	0.273	0.272	0.294	0.299	0.307
	(0.265 - 0.282)	(0.264 - 0.279)	(0.286 - 0.301)	(0.292 - 0.307)	(0.300 - 0.315)
LSM 7	0.097	0.099	0.108	0.108	0.117
	(0.091 - 0.102)	(0.094 - 0.104)	(0.103 - 0.113)	(0.103 - 0.113)	(0.112 - 0.122)
LSM 8	0.065	0.067	0.072	0.073	0.072
	(0.061 - 0.070)	(0.063 - 0.071)	(0.068 - 0.077)	(0.069 - 0.077)	(0.067 - 0.076)
LSM 9	0.080	0.076	0.079	0.079	0.077
	(0.074 - 0.086)	(0.072 - 0.081)	(0.075 - 0.084)	(0.074 - 0.084)	(0.073 - 0.082)
LSM 10	0.065	0.060	0.055	0.060	0.053
	(0.059 - 0.072)	(0.056 - 0.065)	(0.051 - 0.060)	(0.055 - 0.064)	(0.049 - 0.057)
Province: (Proportion)					
Western Cape	0.122	0.122	0.116	0.118	0.117
	(0.116 - 0.129)	(0.117 - 0.126)	(0.112 - 0.121)	(0.114 - 0.123)	(0.112 - 0.122)
Eastern Cape	0.094	0.092	0.097	0.096	0.095
	(0.089 - 0.100)	(0.089 - 0.096)	(0.094 - 0.101)	(0.093 - 0.100)	(0.092 - 0.099)
Northern Cape	0.021	0.021	0.020	0.021	0.021
	(0.019 - 0.022)	(0.020 - 0.022)	(0.019 - 0.022)	(0.020 - 0.023)	(0.020 - 0.022)
Free State	0.060	0.059	0.058	0.056	0.056
	(0.056 - 0.064)	(0.057 - 0.061)	(0.055 - 0.060)	(0.054 - 0.058)	(0.053 - 0.058)
KwaZulu-Natal	0.159	0.158	0.161	0.168	0.169
	(0.152 - 0.166)	(0.153 - 0.163)	(0.156 - 0.166)	(0.163 - 0.173)	(0.164 - 0.175)
North West	0.072	0.073	0.075	0.072	0.072
	(0.067 - 0.077)	(0.069 - 0.076)	(0.072 - 0.079)	(0.068 - 0.075)	(0.069 - 0.075)
Gauteng	0.305	0.308	0.303	0.299	0.300
	(0.292 - 0.318)	(0.301 - 0.315)	(0.297 - 0.310)	(0.292 - 0.306)	(0.292 - 0.307)
Mpumalanga	0.076	0.075	0.074	0.076	0.077
	(0.071 - 0.081)	(0.073 - 0.078)	(0.072 - 0.077)	(0.073 - 0.079)	(0.074 - 0.080)
Limpopo	0.092	0.091	0.094	0.093	0.093
	(0.087 - 0.096)	(0.088 - 0.095)	(0.092 - 0.097)	(0.090 - 0.096)	(0.090 - 0.096)

Notes: Data from Statistics South Africa's General Household Survey, 2014-2018, restricted to households falling into LSMs 4-10. This table calculates serves as a comparison to the sample characteristics of the Kantar Europanel presented in Table 1.

Supplemental Table S3: Beverage types by tax status and tariff codes

HPL Taxable beverage type	Description	HPL Item	Tariff Subheading
Carbonates	Carbonated soft drinks with caloric sweeteners	191.07.05<br>191.07.10	2202.10.10<br>2202.10.90
Fruit drinks & nectars	Ready-to-drink fruit flavoured drinks with no fruit juice, ready-to-drink fruit flavoured drinks with juice, nectars, syrups and powders (reconstituted)	191.05.05<br>191.05.10<br>191.07.25<br>191.07.90	2106.90.20<br>2106.90.22<br>2202.99.20<br>2202.99.90
Other Taxable	Sports drinks, energy drinks, flavoured waters, teas, non-dairy milks/beverages with caloric sweeteners, dairy blend drinks with caloric sweeteners, hot chocolate, malt drinks, flavour straws, aloe drinks, mageu, non-alcoholic beers with caloric sweeteners (ready-to drink and reconstituted)	191.05.05<br>191.07.05<br>191.07.10<br>191.07.25<br>191.07.90<br>191.01.05<br>191.02.05<br>191.05.15<br>191.07.15<br>191.07.20	2106.90.20<br>2202.10.10<br>2202.10.90<br>2202.99.20<br>2202.99.90<br>1806.10.05<br>1901.90.15<br>2106.90.69<br>2202.91.20<br>2202.91.90
**HPL Non-taxable beverage type**	**Description**	**HPL Item**	**Tariff Subheading**
Milk	Milks, dairy drinks and non-dairy milks/beverages without caloric sweeteners	N/A	0401, 0402, 0403, 0404
Bottled Water	Flavored or unflavored waters without caloric sweeteners	N/A	2201.10, 2201.90, 2201
100% Juice	100% juice & coconut water	N/A	2009, 2201.90
Other HPL Non-taxable non-alcoholic drinks	Unsweetened carbonates, flavored and unflavored teas & coffees and coffee substitutes	N/A	2201.1, 0901, 0902, 0903, 2101
Alcohol	Alcoholic beers, wines, cider, spirits, pre-mixed drinks, homebrew kits	N/A	2203, 2204, 2205, 2206, 2207, 2208

Supplemental Table S4: Survey weighted purchases by beverage types

	HPL Taxable Beverages			HPL Non-taxable Beverages				
	Carbonates	Fruit drinks & Nectars	Other Taxable	Milks	Bottled Water	100% Fruit Juices	Other HPL non-taxable non-alcoholic drinks	Alcohol
	Mean (SE)	Mean (SE)	Mean (SE)	Mean (SE)	Mean (SE)	Mean (SE)	Mean (SE)	Mean (SE)
Panel A: Mean Daily Sugar (grams/capita/day)								
Pre-announcement	13.97	1.00	1.28	1.57	0.00	0.98	0.07	0.09
	(0.22)	(0.02)	(0.04)	(0.02)	(0.00)	(0.02)	(0.01)	(0.01)
Pre-implementation, Post-announcement	12.03 [A]	1.04	1.2	1.68 [A]	0	1.18	0.07	0.12
	(0.20)	(0.02)	(0.03)	(0.03)	(0.00)	(0.03)	(0.00)	(0.01)
Post-implementation	8.73 [A,B]	0.82 [A,B]	1.08 [A]	1.54	0	1.31 [A]	0.06	0.18
	(0.19)	(0.02)	(0.03)	(0.04)	(0.00)	(0.04)	(0.00)	(0.02)
Panel B: Mean Daily Calories (kcal/capita/day)								
Pre-announcement	59.08	4.62	6.51	21.37	0	4.66	0.39	18.67
	(0.89)	(0.10)	(0.18)	(0.34)	(0.00)	(0.11)	(0.03)	(0.57)
Pre-implementation, Post-announcement	51.21 [A]	4.83	6.41	23.02	0	5.47	0.38	20.33
	(0.85)	(0.10)	(0.13)	(0.38)	(0.00)	(0.12)	(0.02)	(0.64)
Post-implementation	36.89 [A,B]	3.61 [A,B]	5.96	22.74	0	5.82 [A]	0.37	16.12
	(0.81)	(0.11)	(0.15)	(0.56)	(0.00)	(0.17)	(0.02)	(0.67)
Panel C: Mean Daily Volume (mL/capita/day)								
Pre-announcement	133.3	55.33	330.36	39.81	5.48	9.75	173.73	47.91
	(2.02)	(2.11)	(4.60)	(0.63)	(0.24)	(0.23)	(4.51)	(1.56)
Pre-implementation, Post-announcement	117.84 [A]	55.03	319.29	42.94	8.88	11.75	197.92	42.7
	(1.93)	(1.70)	(4.34)	(0.71)	(0.37)	(0.26)	(5.66)	(1.50)
Post-implementation	94.99 [A,B]	45.01 [A,B]	303.4 [A]	42.72	9.61	13.74 [A]	210.51 [A]	26.78 [A,B]
	(2.06)	(1.69)	(5.57)	(1.17)	(0.45)	(0.40)	(7.69)	(1.40)

Notes: Mean values are survey weighted to be representative of 13.7 million South African households with LSM ≥4. [A] denotes statistically significantly different from pre-announcement values at p<0.01; [B] denotes statistically different from pre-implementation/post-announcement values at p<0.01. Data covers household purchases from January 2014–March 2019 South Africa Europanel (the legal entity of which is Kantar UK Ltd), a joint venture of GfK and Kantar Worldpanel. SE= Standard Error

Supplemental Table S5: Regression Coefficients

	Sugar (g/capita/day)		Calories (kcal/capita/day)		Volume (mL/capita/day)	
	Taxable	*Non-Taxable*	*Taxable*	*Non-Taxable*	*Taxable*	*Non-Taxable*
Pre x Time	0.129***	0.0266***	0.613***	0.424***	2.874***	1.957***
	(0.0232)	(0.00499)	(0.0975)	(0.0858)	(0.688)	(0.609)
Post Announcement	8.215***	0.243	38.34***	18.60***	170.7***	98.20***
	(0.991)	(0.245)	(4.205)	(3.893)	(32.60)	(30.31)
Post Announcement x Time	-0.220***	0.0167***	-1.002***	-0.122	-4.140***	-1.122
	(0.0244)	(0.00632)	(0.104)	(0.0987)	(0.832)	(0.784)
Post Implementation	8.527***	-0.0248	37.27***	2.172	-20.36	-122.8
	(2.536)	(0.796)	(10.78)	(10.45)	(94.18)	(93.84)
Post Implementation x Time	-0.215***	0.0157	-0.933***	0.127	-0.0446	3.259**
	(0.0443)	(0.0142)	(0.189)	(0.183)	(1.658)	(1.659)
Constant	20.78***	4.828***	97.31***	58.55***	819.9***	588.5***
	(1.785)	(0.655)	(9.650)	(5.810)	(115.1)	(75.45)
Observations	113,653	113,653	113,653	113,653	113,653	113,653
R-squared	0.462	0.372	0.475	0.445	0.424	0.356
AIC	1.021e+06	702881	1.350e+06	1.336e+06	1.818e+06	1.806e+06
BIC	1.021e+06	703151	1.350e+06	1.336e+06	1.819e+06	1.807e+06

Notes: Data covers household purchases from January 2014–March 2019 South Africa Europanel (the legal entity of which is Kantar UK Ltd), a joint venture of GfK and Kantar Worldpanel. Regressions adjust for: household characteristics (household size, number of adults, life-cycle stage, total food and non-food spending) and provincial characteristics(CPI). Robust standard errors in parentheses. *** p<0.01, ** p<0.05, * p<0.1

Supplemental Table S6: Non-Taxable Beverage Purchases Relative to Pre-Trend by LSM

	**Estimated Purchase**	**Pre-Trend counterfactual purchases**	**Difference**	**Relative Difference**
	Mean (CI)	Mean (CI)	Mean (CI)	%
Panel A: Lower LSM (4-6)				
Pre-Implementation, Post-Announcement Period				
Sugar (g/capita/day)	2.73	2.85	-0.12	-4.21%
	(2.46, 3.00)	(2.26, 3.43)	(-0.62, 0.38)	
Calories (kcal/capita/day)	51.99	61.54	-9.55	-15.52%
	(45.01, 58.97)	(48.15, 74.93)	(-19.44, 0.33)	
Volume (mL/capita/day)	278.62	323.35	-44.73	-13.83%
	(241.20, 316.05)	(240.13, 406.58)	(-118.69, 29.23)	
Post-Implementation Period				
Sugar (g/capita/day)	2.38	3.02	-0.63	-20.86%
	(2.01, 2.76)	(2.04, 4.00)	(-1.55, 0.28)	
Calories (kcal/capita/day)	40.82	71.17	-30.35	-42.64%
	(33.80, 47.83)	(51.05, 91.29)	(-49.64, -11.07)	
Volume (mL/capita/day)	260.82	343.36	-82.54	-24.04%
	(219.39, 302.25)	(204.51, 482.22)	(-213.86, 48.78)	
Panel B: Higher LSM (7-10)				
Pre-Implementation, Post-Announcement Period				
Sugar (g/capita/day)	3.55	3.69	-0.14	3.69&
	(3.28, 3.82)	(3.13, 4.25)	(-0.62, 0.34)	
Calories (kcal/capita/day)	50.16	45.67	4.49	9.83%
	(45.70, 54.62)	(37.35, 53.99)	(-3.49, 12.47)	
Volume (mL/capita/day)	306.47	303.16	3.31	1.09%
	(280.70, 332.23)	(252.58, 353.73)	(-43.77, 50.39)	
Post-Announcement Period				
Sugar (g/capita/day)	3.54	4.19	-0.64	-15.27%
	(3.09, 3.99)	(3.26, 5.11)	(-1.57, 0.28)	
Calories (kcal/capita/day)	49	48.74	0.26	0.53%
	(43.64, 54.36)	(35.25, 62.22)	(-12.86, 13.39)	
Volume (mL/capita/day)	315.73	334.68	-18.95	-5.66%
	(278.24, 353.23)	(248.53, 420.83)	(-100.97, 63.08)	

Notes: CI= 95% Confidence Interval; LSM = living standards measure. Mean values are survey weighted to be representative of 13.7 million South African households with LSM ≥4. Data covers household purchases from January 2014–March 2019 South Africa Europanel (the legal entity of which is Kantar UK Ltd), a joint venture of GfK and Kantar Worldpanel.

REFERENCES

1. Colchero MA, Popkin BM, Rivera JA, Ng SW. Beverage purchases from stores in Mexico under the excise tax on sugar sweetened beverages: observational study. BMJ. 2016;352.

2. Colchero MA, Rivera-Dommarco J, Popkin BM, Ng SW. In Mexico, Evidence Of Sustained Consumer Response Two Years After Implementing A Sugar-Sweetened Beverage Tax. Health Affairs. 2017;36(3):564-71.

3. Caro JC, Corvalán C, Reyes M, Silva A, Popkin B, Taillie LS. Chile’s 2014 sugar-sweetened beverage tax and changes in prices and purchases of sugar-sweetened beverages: An observational study in an urban environment. PLOS Medicine. 2018;15(7):e1002597.

4. Nakamura R, Mirelman AJ, Cuadrado C, Silva-Illanes N, Dunstan J, Suhrcke M. Evaluating the 2014 sugar-sweetened beverage tax in Chile: An observational study in urban areas. PLOS Medicine. 2018;15(7):e1002596.
